# Invited Commentary: Undiagnosed and Undertreated—the Suffocating Consequences of the Use of Racially Biased Medical Devices During the COVID-19 Pandemic

**DOI:** 10.1093/aje/kwad019

**Published:** 2023-01-25

**Authors:** Marie V Plaisime

**Keywords:** clinical trials, COVID-19, Food and Drug Administration, hypoxia, pulse oximetry, racial bias, racism, skin pigmentation

## Abstract

While medical technology is typically considered neutral, many devices rely upon racially biased algorithms that prioritize care for White patients over Black patients, who may require more urgent medical attention. In their accompanying article, Sudat et al. (*Am J Epidemiol*. 2023;XXX(XX):XXX–XXX) document striking inaccuracies in pulse oximeter readings among Black patients, with significant clinical implications. Their findings suggest that this resulted in racial differences in delivery of evidence-based care during the coronavirus disease 2019 (COVID-19) pandemic, affecting admissions and treatment protocols. Despite the medical community’s growing awareness of the pulse oximeter’s significant design flaw, the device is still in use. In this article, I contextualize Sudat et al.’s study results within the larger history of racial bias in medical devices by highlighting the consequences of the continued underrepresentation of diverse populations in clinical trials. I probe the implications of racially biased assessments within clinical practice and research and illustrate the disproportionate impact on patients of color by examining 2 medical tools, the pulse oximeter and pulmonary function tests. Both cases result in the undertreatment and underdiagnosis of Black patients. I also demonstrate how the social underpinnings of racial bias in medical technology contribute to poor health outcomes and reproduce health disparities, and propose several recommendations for the field to rectify the harms of racial bias in medical technology.

This article is linked to "Racial Disparities in Pulse Oximeter Device Inaccuracy and Estimated Clinical Impact on COVID-19 Treatment Course" (https://doi.org/10.1093/aje/kwac164).

## Abbreviations

COVID-19: coronavirus disease 2019
FDA: Food and Drug Administration
NIH: National Institutes of Health

***Editor’s note:** The opinions expressed in this article are those of the author and do not necessarily reflect the views of the* American Journal of Epidemiology.

At the height of the coronavirus disease 2019 (COVID-19) pandemic, the Centers for Disease Control and Prevention proposed clinical protocols to triage the deployment of different therapeutic medical interventions. As a result, the use of medical technology increased significantly at home and in hospital settings. Medical staff relied on information provided by patients (i.e., symptoms) and medical tools (e.g., pulse oximeters, thermometers) to determine hospital and intensive care unit admissions and the distribution of supplemental oxygen therapies, including mechanical ventilation. While medical technology is typically considered neutral, many devices rely upon racially biased algorithms that prioritize care for White patients over care for Black patients, who may require more urgent medical attention.

The article by Sudat et al. (1) in this issue of the *Journal* documents striking inaccuracies in pulse oximeter readings among Black patients. The authors conducted retrospective cohort analyses using electronic health records and investigated pulse oximetry measurements of oxygen saturation in self-identified non-Hispanic Black and non-Hispanic White patients. They found that differences in oxygen saturation measurements influenced hospital admissions, treatment delays, health-care delivery, and hospital readmission rates in both patient groups. They found that oximeters overestimated oxygen saturation measurements for Black patients, influencing care, and adding to the literature documenting admission and treatment delays, inequitable health-care delivery, and disparate hospital readmission rates.

It is critical to examine the totality of how technology is used in clinical encounters to produce and reproduce health inequities. Our reliance on biased instruments and measurements has led to errors, including our ability to detect hypoxia effectively. This has several implications for patients, including treatment delays and unnecessary intubation (2). Oximeter readings overestimate oxygen levels in patients with darker skin pigmentation. Research shows that patients with darker skin pigmentation are 3 times more likely to experience pulse oximeter measurement errors (3, 4). Despite the medical community’s awareness of the pulse oximeter’s consequential design flaw, the device is still in use and provides inaccurate measurements for Black and Brown patients.

In this commentary, I contextualize Sudat et al.’s study results within the larger history of racial bias in medical devices. Specifically, I describe pulse oximeter devices and then provide historical and social context by highlighting the roles of the Food and Drug Administration (FDA), the National Institutes of Health (NIH), and the continued underrepresentation of diverse populations in clinical trials. I probe the implications of racially biased assessments within clinical practice and research and illustrate the disproportionate impact on patients of color by examining 2 medical tools, the pulse oximeter and pulmonary function tests. Both cases result in the undertreatment and underdiagnosis of Black patients. I also demonstrate how the social underpinnings of racial bias in medical technology contribute to poor health outcomes and reproduce health disparities, and I propose several recommendations for the field to rectify the harms of racial bias in medical technology.

## COVID-19 AND PATIENTS OF COLOR

**Figure 1:**
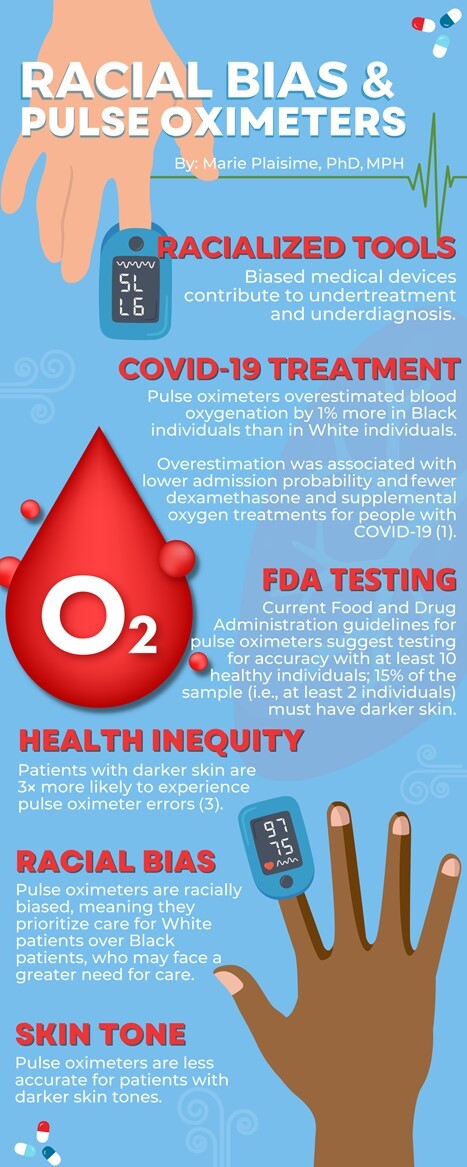
Important factors related to pulse oximeters. COVID-19, coronavirus disease 2019; FDA, Food and Drug Administration.

As of September 2022, over 1 million patients had died from COVID-19 in the United States (5). Research shows that the Black, Hispanic, American Indian/Alaska Native, and Native Hawaiian/Pacific Islander populations have disproportionately experienced higher rates of COVID-19 morbidity and mortality, with Black patients being twice as likely to die from COVID-19 as White patients (5). Considering the unequal impact, Black patients have been penalized twice during the COVID-19 pandemic (Figure 1). First, pulse oximeter readings overestimate oxygen levels in Black patients, thereby reducing their likelihood of receiving necessary supplemental oxygen. For example, Fawzy et al. (6) found that pulse oximeter measurements overestimated oxygen saturation for 1.2% of Black patients and 1.1% of Hispanic patients. This may directly affect unrecognized or delayed recognition of a patient’s eligibility to receive specific COVID-19 therapies. Second, during COVID-19 recovery, Black patients’ lung function may have been spuriously overestimated due to race-based calibration of spirometry (7).

### Race-based medicine and race correction

The concepts of race, genetics, and skin tone are not synonymous. “Race” is a social and political construct with no inherent biological or genetic basis. Furthermore, it does not accurately reflect skin hue, pigmentation, or human biological diversity (8). Racial assignments are products of racism, prejudice, and discrimination. These typologies are nuanced and vary by factors such as physical features (e.g., hair texture), and racial self-identification often echoes complex cultural experiences and group dynamics (9). Evidence shows that genetic history, rather than race, provides more accurate information for specific conditions. However, since race and genetics are often conflated and used interchangeably, incorporating race for diagnosis and treatment causes harm (10). Race correction is the practice of adjusting medical calculations to account for race (11). For decades this was standard practice in several medical devices and tools, including the estimated glomerular filtration rate, vaginal birth after cesarean delivery, and pulmonary function tests (12).

### Pulmonary function tests

Until recently, the pulmonary function test adjusted for race, with decreased predicted lung function capacity for Blacks. Spirometer measurements are often adjusted for Black and Asian patients, because race is often assumed to be a biological trait (13). The notion that lung capacity differed between Black and White patients *emanated* from American chattel slavery, where individuals such as Cartwright endorsed ideas that “lung capacity” deficiencies existed in Black bodies and that race was an essential biological factor (14). Such adjustments do not consider the structural factors that affect lung capacity, such as environmental racism, occupation, and proximity to carcinogenic materials. However, due to scientific racism and race-based practices such as race correction, many patients face suffocating consequences, which include undertreatment and deprioritization for care.

**Figure 2:**
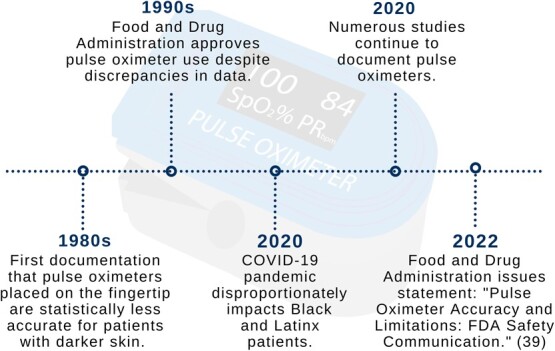
Historical timeline of pulse oximetry fingertip accuracy. COVID-19, coronavirus disease 2019; FDA, Food and Drug Administration.

## THE DEVICE: PULSE OXIMETERS

Oximeters were initially developed in World War II to measure ear oxygen saturation among airline pilots; since then, the tool has evolved (15, 16). Pulse oximeters are small noninvasive medical devices that are placed on the fingertip to rapidly measure peripheral arterial oxygen saturation in the blood. They typically assess oxygen saturation using spectrophotometry. Pulse oximeters use 2 or more wavelengths to measure peripheral oxygen saturation (8, 17). Light passes through the fingertip, yielding a measured light output that varies with skin tone. This is because the melanocyte, a cell responsible for producing skin, hair, and eye pigmentation, highly absorbs light changes and thus limits the penetration of light into tissue (18). Pulse oximeters are often calibrated using the Fitzpatrick Scale, which contains 6 skin tone types ranging from type 1 (“light, pale”) to type 6 (“darkest brown”) (19). The limited and rigid options available in this scale contribute to biased outputs (20, 21).

Within the medical setting, pulse oximeter devices determine 1) whether a patient should receive supplemental oxygen and 2) the amount of oxygen a patient should receive. These devices are used to evaluate many conditions, including asthma, pneumonia, anemia, congenital heart disease, and lung cancer (22), and they are especially useful in testing for occult hypoxemia (i.e., low oxygen levels). Research shows that detection of severe hypoxemia is less accurate in patients with darker skin tones because peripheral oxygen saturation is overestimated (23, 24). For example, Henry et al. (25) found that Black patients experienced more occult hypoxemia than White patients, and this was associated with increased mortality.

Pulse oximeter inaccuracy is a global concern. Oximeter measurement errors harm Black patients and all patients with darker skin tones. While Sudat et al. exclusively considered non-Hispanic White and non-Hispanic Black patients (1), other studies have found differences in measurement among Chinese, Malay, and Indian patients (26). Sudat et al.’s use of race/ethnicity (i.e., non-Hispanic Black and non-Hispanic White) highlights a greater need to investigate the impact of measurement error across the spectrum of skin pigmentation.

## HISTORICAL AND CONTEMPORARY CONTEXT OF DEVICE DEVELOPMENT AND APPROVAL

Inaccuracies in the measurement of fingertip oxygen saturation have been made apparent since the 1970s (27) (Figure 2). In 1987, Cecil et al. (28) found that pulse oximeters were statistically less accurate for patients with darker skin. In 1990, Jubran and Tobin (29) documented reliable peripheral oxygen saturation measurements for White patients receiving mechanical ventilation and overestimated measurements for Black patients, which was also commonly associated with significant hypoxemia. Despite demonstrated evidence of inaccuracy, pulse oximeters remained in use in clinical settings. It is crucial to connect the biased performance of pulse oximeters to their validation via clinical trials. Historically, clinical trials have been noninclusive, and validation studies have included inadequate numbers of Black patients to determine measurement errors. The NIH’s lack of success in diversifying clinical trials has had painful and unjust long-term complications.

### The FDA

Two significant responsibilities of the FDA include “protecting the public health by assuring the safety, efficacy, and security of human and veterinary drugs, biological products, [and] medical devices” (30) and “helping the public get the accurate, science-based information they need to use medicines and foods to maintain and improve their health” (31). The pulse oximeter is an FDA-approved device, and for decades, it has been permitted to be sold without adequate testing in diverse clinical populations. Instead, the FDA suggests premarket testing of medical devices, which are generally approved through a 510(k) provision, which does “not require clinical trials or manufacturing inspections to demonstrate safety and efficacy” (32, pp. 1006–1007). A company must prove that its device is “substantially equivalent” to (32, p. 1007) and aligns with other FDA devices approved before May 1976, because Congress did not give the FDA the authority to regulate all medical devices until 1976. Current FDA guidelines for pulse oximeters suggest testing the device for accuracy with at least 10 healthy individuals; 15% of the sample (i.e., at least 2 individuals) must have darker skin (18).

The NIH and FDA work in concert to “[advance] public health by promoting the translation of basic and clinical research findings into medical products and therapies” (33). The NIH manages and supports medical research such as clinical trials to support innovative research; the FDA regulates quality control and effectiveness to approve medical products. In 1993, congress passed the NIH Revitalization Act, which encouraged the increased inclusion of women and historically marginalized racial and ethnic groups in the design and execution of clinical trials (34). Since the invention of the device in 1974, data used to calibrate the pulse oximeter have been based on healthy self-identified White volunteers and light skin tones. This should be of no surprise as, historically, clinical trials have been noninclusive (35).

### Current practices and other devices

Still, in 2023, most medical devices in the United States are tailored for White bodies. For example, other devices, including forehead thermometers and tools for identifying veins, may also be inaccurate for patients with more melanin, documenting inaccuracies in output measurements (36). Both were tested primarily on young and middle-aged men of European descent (8). National reports indicate that Black patients represent only 5% of overall testing samples (37) in NIH clinical trials of devices across several health outcomes. For example, in clinical trials on cardiometabolic drugs, Black patients represent 4% of participants (38). This also highlights more significant issues related to misclassifications and misinterpretations of race and ethnicity (39, 40).

Recently, the FDA announced plans to change pulse oximeter standards, and it launched several initiatives to improve strategies for better calibrating the device to perform equally across all skin tones and to promote diversity within clinical trials on the device (41). However, to date, no standards have been approved, and providers have been encouraged to proceed cautiously. Where does this leave researchers, epidemiologists, clinicians, public health practitioners, and most importantly, patients?

## WHAT IS THE ROLE OF EPIDEMIOLOGY?

Epidemiologists are well positioned to investigate the processes that create and sustain racial bias within our larger societal systems, interpersonal interactions, and the tools used within medicine. The work to eliminate bias from the clinical encounter requires assistance at all levels. On a large scale, researchers have explored how bias influences health outcomes, such as the influence of residential segregation and discriminatory housing policies (42). On an individual level, scholars have also advanced our understanding of how racial bias across different domains (e.g., medicine, policing, employment and hiring practices) impacts health outcomes (43). We must now investigate bias on a granular level; the pulse oximeter is a perfect example. Epidemiologists and other public health disciplines must rigorously examine bias and the hidden biases embedded in medicine. There are likely many other sources of hidden bias contributing to inequitable treatment that have yet to be discovered. The continued use of nonrepresentative data is detrimental.

Considering the large-scale impact on patients with darker skin tones, we must assess regulations and policies concerning the manufacturing, testing, marketing, and distribution of pulse oximeters. The FDA must require sufficient skin tone representation in their new guidelines to allow for sufficiently powered statistical tests. We must continue to evaluate the impact on patients and the potential impact on various clinical outcomes, including morbidity, mortality, and overuse or underuse of procedures (i.e., intubation) for different patients. Designing inclusive tools improves health-care quality for all. A commitment to diversity and inclusion will support novel strategies to remove and avoid bias within future medical tools and practices.

Measurement inaccuracy is not only a clinical issue but a public health crisis; precision is vital in all research designs. Misclassification error and measurement error compromise reliability and validity; this raises concern about pulse oximeter measurements overall. Given the limited representation and testing, the medical community, researchers, and consumers should be cautious about these measurements. Leading scholars like Dr. Ellis Monk are investigating new ways to expand technology’s color palette. The Monk Skin Tone Scale expands the Fitzpatrick Scale and includes a more inclusive index of scales (44). Additional research is needed to quantify the measurement error across different racial categories and skin pigmentations, not only among Black patients. This requires a reimagination of educational processes across workplace sectors (i.e., social scientists, engineers, marketing) and designers to be inclusive. We must also standardize practices regarding the measurement and reporting of oxygen saturation (20).

Epidemiologists and the public health community can contribute to rectifying racial bias in medicine by considering essential questions:

1. *Consider* the social and historical context of data collection practices (e.g., what historical policies and procedures have influenced your research design?).
2. *Assess* your definition and measure of “race” and “ethnicity.”
3. *Investigate* the original use of race in secondary data (e.g., how were the data on race collected and measured?).
4. *Examine* the impact of flawed data in real-world applications (e.g., use of electronic records in research design).
5. *Incorporate* an equity lens in research design (e.g., who is and is not included?).
6. *Diversify* research design at all stages (e.g., design racially diverse clinical trials and public health interventions with an understanding of structural racism).

## CONCLUSION

As a society, we often believe that technology is benign and neutral; as health practitioners, that our tools are benevolent. On the contrary, many medical devices are racially biased and carry a strong legacy of racism. Historically marginalized groups suffer disproportionality from the remnants of racist policies and structural forms of racism (i.e., segregation, redlining, zoning). Sudat et al.’s novel approach to assessing oxygen saturation emphasizes the critical need to redesign medical devices. Biased tools can misrepresent disease severity and threaten overall health-care quality. As the COVID-19 pandemic continues, scientists, clinicians, epidemiologists, medical practitioners, the NIH, the FDA, and other members of the medical device community must address their role in creating a healthier world, and explicitly improving the accuracy of pulse oximeters is an essential first step. This includes an intentional evaluation of the multidimensional components of bias in health care and clinical practices and investigation of the intersections between race, structural racism (e.g., team and knowledge diversity), and health outcomes. Racially biased tools and inaccuracies within pulse oximeters and pulmonary function tests are not random but are products of racialized historical policies, statistical errors, and research design flaws.
